# Beyond the needle: how clinician expertise affects outcomes in the treatment of hemifacial spasm

**DOI:** 10.1016/j.prdoa.2025.100383

**Published:** 2025-08-06

**Authors:** Melissa Luque-Llano, Maria Angelica-Coronel, Yulexy T. Alvarado-Vanegas, Floralinda García Puello, Laura Arzuza Ortega, Gustavo B. Vincos

**Affiliations:** a Department of Neurology Universidad Simón Bolívar, Health sciences faculty, Barranquilla 080001, Colombia; b Universidad Simón Bolívar, Health Sciences Faculty, Barranquilla 080001, Colombia; c Department of Neurology, Movement Disorders Unit – Hospital Occidente de Kennedy. Bogotá 110821, Colombia

**Keywords:** Hemifacial spasm, Botulinum toxin, Adverse effects, Neurologist, Resident

## Abstract

**Background:**

Hemifacial spasm (HFS) is characterized by abnormal contraction of the muscles innervated by the seventh cranial nerve. It is usually benign and diagnosed clinically. The effectiveness of botulinum toxin (BoNT) for its treatment has been well demonstrated. However, adverse events (AEs) resulting from its administration remain a concern, and some patients express reluctance to be treated by resident physicians, even when under expert supervision.

**Objective:**

To determine the association between the level of expertise of the neurology professional and the presence of AEs following BoNT administration in patients with HFS.

**Methods:**

A cross-sectional descriptive study with an analytical phase conducted at Hospital Occidente de Kennedy (Bogotá, Colombia). Out of 88 medical records reviewed, 73 met all inclusion criteria. Data analysis was performed using SPSS software version 22.

**Results:**

A predominance of female patients was observed, accounting for 74 % of the study population. The mean age of symptom onset was 54.5 ± 12.7 years, with a median symptom duration of 9 years. In most cases, the left side was affected (53.4 %). The average number of botulinum toxin applications per patient was 2.6 during the study period. Adverse events occurred in 12.6 % of cases, primarily mild facial paresis (7.9 %), hematoma (2.6 %), and eyelid ptosis (2.1 %)

**Conclusions:**

The results did not show a statistically significant association between the level of expertise and the occurrence of adverse events. The frequency of observed adverse events was similar between the groups. This suggests that the occurrence of adverse events following BoNT administration was comparable regardless of whether the procedure was performed by a neurologist or a resident under supervision.

## Highlights

• BoNT-A injections by supervised residents showed no increased risk of adverse events in HFS patients.
• Adverse event rates were similar between neurologists (12.3%) and supervised residents (12.7%).
• Structured training and supervision ensured safe BoNT-A administration by neurology residents.
• Study supports supervised trainee involvement in HFS care without compromising patient safety.

## 1 Introduction

Hemifacial spasm (HFS) is a movement disorder characterized by unilateral involuntary muscular contractions of the muscles innervated by the facial nerve. First-line treatment consists of injections with botulinum toxin type A (BoNT-A), which is considered a safe and effective long-term therapy for reducing motor symptoms and improving patients' quality of life [[1], [2], [3]].

The true prevalence of hemifacial spasm is difficult to determine, in part due to frequent misdiagnosis stemming from limited awareness of movement disorders among healthcare professionals [1]. In the United States, prevalence has been estimated at approximately 11 cases per 100,000 individuals [[1], [2], [3], [4]], with higher rates reported in Asian populations [4,5]. Currently, no epidemiological studies have evaluated its prevalence in the Colombian population.

HFS is diagnosed clinically. A detailed medical history and comprehensive neurological examination are required to rule out signs of secondary causes. Complementary tools include electromyography (EMG) to exclude facial nerve injuries, and brain magnetic resonance imaging (MRI) to rule out structural lesions or demyelinating diseases of the brainstem [1]. The most frequently affected muscles, in descending order, are the orbicularis oculi (94 %), perioral muscles (63 %), zygomatic muscles (55 %), platysma (33 %), frontalis (24 %), and mentalis (21 %) [5].

BoNT-A administration requires specialized technical skills and precise anatomical knowledge, as adverse effects such as eyelid ptosis, facial paresis, hematomas, and even lagophthalmos have been reported [2,6]. These procedures are commonly performed by neurology residents in training at university hospitals, often under the direct supervision of specialists. This context presents significant challenges for medical training programs, as they must ensure a safe learning curve without compromising patient care [7,8].

In other medical disciplines, the so-called “July effect” has been described, referring to a potential increase in medical errors or complications at the start of the academic year [9]. Although evidence in neurology is limited, this phenomenon has sparked debate about the relationship between practitioner experience and patient safety.

Furthermore, qualitative studies have shown that some patients may experience anxiety or rejection at the idea of being treated by trainees, particularly when the procedures involved are invasive or technically demanding [10].These perceptions not only represent emotional or psychological barriers, but also reflect ethical concerns related to patient autonomy, informed consent, and the perception of fairness and safety in medical care [11].

Despite the extensive evidence supporting the efficacy of botulinum toxin in the treatment of HFS, few studies have evaluated the impact of practitioner experience level on the occurrence of adverse events. This study aims to contribute to this field by exploring the association between injector expertise (neurologist vs. supervised neurology resident) and procedural safety in a real-world academic clinical setting in a movement disorders clinic.

## 2 Methods

A cross-sectional descriptive study with an analytical component was conducted. The study population included patients diagnosed with HFS who were treated at Hospital Occidente de Kennedy in Bogotá, Colombia, between January 1st and December 31st of 2024. Participants were selected based on clinical records of patients with primary HFS who received botulinum toxin treatment and were evaluated in the Movement Disorders outpatient clinic at the same hospital.

A total of 88 patients were diagnosed with primary HFS by the same fellowship-trained movement disorder neurologist (GBV1), with over 10 years of experience, and received treatment with botulinum toxin type A (BoNT-A) during the study period. Of these, 73 patients met all inclusion criteria (19 men and 54 women).

A total of 191 botulinum toxin applications were analyzed in these 73 patients. A comparative analysis was performed to evaluate the occurrence of adverse events (AEs) following injections administered either by the movement disorder specialist or by supervised neurology residents.

Patients were included in the study if they met the following inclusion criteria:

• Age ≥ 18 years and evaluated in the outpatient Neurology/Movement Disorders clinic.
• Diagnosis of HFS made by a neurologist specialized in movement disorders (HFS diagnosis is established based on clinical evidence of unilateral, involuntary tonic and/or clonic contractions of muscles innervated by the facial nerve).
• Patients with bilateral HFS were included if symptom onset was not simultaneous and contractions were asynchronous.
• Received at least one treatment with onabotulinumtoxinA (Botox®) between January 1, 2024, and December 31, 2024.

Exclusion criteria were as follows:

• Other facial dyskinesias (e.g., blepharospasm, oromandibular dystonia, facial tics, myokymia, hemimasticatory spasm, or functional neurological disorders).
• Secondary HFS, defined by a history of trauma or facial nerve injury on the affected side, prior facial paralysis, facial nerve palsy signs on neurological examination, or MRI findings suggestive of a secondary cause.
• Synkinesis without involuntary facial movements.
• Treatment with botulinum toxin formulations other than onabotulinumtoxinA (Botox®).

The therapeutic intervention consisted of subcutaneous botulinum toxin injections administered based on the patient’s clinical presentation. Injections were performed either by a movement disorder neurologist with over 10 years of experience or by third- and fourth-year neurology residents under direct supervision (PGY-3 in their second semester and PGY-4 in their first semester). Six residents participated in total. They received prior structured training, including theoretical instruction on BoNT-A use in neurological conditions and supervised practice using anatomical facial models before clinical application.

All residents had previously performed BoNT-A procedures in other clinical contexts (e.g., spasticity, migraine), but the injections for hemifacial spasm were conducted under direct supervision of a movement disorder specialist. This training strategy ensured standardized procedures and minimized clinical risk.

All procedures followed a standardized institutional protocol, which included defined techniques, dilution, and injection sites for managing HFS. The dilution used was 1 mL per 50 U vial and 2 mL per 100 U vial of onabotulinumtoxinA (Botox®). The most frequently injected muscles were the orbicularis oculi, zygomaticus, mentalis, and platysma. The most common injection scheme included four points in the orbicularis oculi (2.5 U each), one point in the zygomaticus (5 U), one in the mentalis (5 U), and two in the platysma (2.5 U each), totaling 25 U per hemiface. This technical approach remained consistent throughout the study, applied by both specialists and supervised residents, reducing potential bias due to technical variability.

Injection patterns were modified according to each patient's response in subsequent treatment sessions during the one-year follow-up. Follow-up intervals were individualized based on treatment effectiveness, with an intended average interval of 3 to 6 months. The timing between sessions was determined either by clinical improvement observed during scheduled evaluations or by symptom recurrence reported by the patient. In addition, standardized video recordings were obtained prior to each injection and at a follow-up visit approximately one month after the procedure. These videos were used to objectively document treatment response and motor symptom progression over time.

Adverse events were recorded when reported either by the neurologist or by the patient. Neurologist-reported AEs had to be directly observed and documented in the medical record. Patient-reported AEs could be communicated during follow-up visits. For analytical purposes, adverse events included ptosis, facial paresis, and hematoma.

### 2.1 Data analysis

Data were analyzed using SPSS software version 22, licensed to Universidad Simón Bolívar. Descriptive and inferential statistical methods were used to compare patient characteristics and the occurrence of adverse events following botulinum toxin administration. Quantitative variables were expressed as mean ± standard deviation (SD) or median and interquartile range (IQR), depending on data distribution. Categorical variables were reported as absolute frequencies and percentages.

Second, patients with and without adverse events following BoNT-A injection were compared. The Student’s *t*-test was used for normally distributed continuous variables, and the Mann-Whitney *U* test was used for non-parametric variables. Categorical variables were compared using the chi-square test or Fisher’s exact test, as appropriate.

Third, the association between the occurrence of adverse events and the injector’s level of expertise (neurologist vs. resident) was assessed using a binary logistic regression model, reporting odds ratios (ORs) with corresponding 95 % confidence intervals (95 % CI).

Finally, a stratified analysis was performed by type of adverse event to explore specific associations between each complication and the injector type. For this, 2 × 2 contingency tables were constructed for each event, and crude ORs were estimated using binary logistic regression.

A p-value of < 0.05 was considered statistically significant.

## 3 Results

Table 1 presents the sociodemographic and clinical characteristics of patients with hemifacial spasm treated with botulinum toxin. Most affected individuals were female, with 54 cases accounting for 74 % of the sample (female-to-male ratio of 2.8:1). The mean age was 64.6 ± 13 years, and the condition predominantly affected the left side of the face (53.4 %). The mean age at symptom onset was 54.5 ± 12.7 years, with the youngest patient being 26 years old and the oldest 100 years old. The median symptom duration was 9 years, with an interquartile range (IQR) of 6.9 years, indicating a marked chronicity of the disease with moderate variability among cases.

**Table 1:** Sociodemographic and clinical characteristics of patients with hemifacial spasm treated at Hospital Occidente de Kennedy (Bogotá, Colombia).

**Variables**	**Categories**	**Frequency**	**Percentage / Mean ± SD**
**Sociodemographic Characteristics**			
**Sex**	Male	19	26.0 %
	Female	54	74.0 %
**Age (years)**	Mean ± SD	64.6	± 13.1
**Clinical Characteristics**			
**Lesion laterality**	Bilateral	2	2.7 %
	Right	33	44.0 %
	Left	38	53.4 %
**Age at symptom onset (years)**	Mean ± SD	54.5	± 12.7
**Symptom duration (years)**	Mean ± SD	9	± 6.9

Of the 191 applications, the median dose administered was 25 units (IQR = 13). The median follow-up time for cases was 4 years at the movement disorders clinic (IQR = 5). Residents performed 70.2 % of the applications, and neurologists 29.8 %. Regarding the injector: 8 patients (10 %) received injections exclusively from a neurologist, 33 patients (45.2 %) from residents, and 32 patients (43.8 %) received injections from both (mixed administration). The average number of botulinum toxin injections during the study period was 2.6 per patient (IQR = 1.1). Adverse events occurred in 12.6 % of applications, mainly mild facial paresis (7.9 %), hematoma (2.6 %), and eyelid ptosis (2.1 %). See Table 2.

**Table 2:** Characteristics of botulinum toxin treatment and adverse events.

**Variables**	**Categories**	**N = 191 applications**	**Percentage**
**Follow-up time at MD clinic, (years)**	Median (IQR)	4 (5)	
**Toxin dose (units)**	Median (IQR)	25 (13)	
**Administering professional**	Neurologist	57	29.8 %
	Resident	134	70.2 %
**Number of applications during the study period (2024)**	Median (IQR)	2.6 (1.1)	
**Presence of adverse events**	No	167	87.4 %
	Yes	24	12.6 %
**Type of adverse event**	Hematoma	5	2.6 %
	Facial paresis	15	7.9 %
	Ptosis	4	2.1 %

Table 3 presents the comparison between botulinum toxin applications with and without adverse events. No statistically significant differences were found between clinical or demographic variables and the occurrence of adverse events (all p-values > 0.05). This includes sex, age, laterality, disease duration, follow-up time, administered dose, injector’s level of expertise, and number of applications.

**Table 3:** Comparison Between Study Variables and the Presence of Adverse Events.

**Variables**	**Without Adverse Event (n = 167)**	**With Adverse Event (n = 24)**	**p**
**Sex**			
**Female**	134(80,2%)	20(83,3%)	1,0***
**Male**	33(19,8%)	4(16,7%)	
**Age in years, Mean ± SD**	64,3 ± 15,5	65,5 ± 8,6	**0,7
**Laterality**			
**Bilateral**	5(3 %)	0	
**Left**	86(51,5%)	16(66,7%)	O,44***
**Right**	76(45,5%)	8(33,3%)	
**Age at Symptom Onset, Mean ± SD**	54 ± 15,5	55 ± 7,3	**0,6
**Symptom Duration, Median (IQR)**	9(6)	8,5(9)	*0,96
**Follow-up Time in Years, Median (IQR)**	4(6)	5(6)	*0,31
**Botulinum Toxin Dose – Units, Median (IQR)**	25(10)	25(17)	*0,64
**Injecting Professional**			
**Neurologist**	50(29,9%)	7(29,2%)	*1,0
**Resident**	117(70,1%)	17(70,8%)	
**Number of Applications, Median (IQR)**	3(2)	3(2)	*0,13

*Mann-Whitney *U* test.

**Student's *t*-test.

***Fisher-Freeman-Halton exact test.

Table 4 shows the association between the expertise level of the professional administering the botulinum toxin (neurologist vs. resident) and the occurrence of adverse events. The proportion of adverse events was 12.3 % in the neurologist group (7 of 57 applications) and 12.7 % in the resident group (17 of 134 applications). The estimated odds ratio (OR) was 1.04 (95 % CI: 0.41–2.66), suggesting no statistically significant association between the injector's expertise level and the likelihood of developing adverse events.

**Table 4:** Association between the neurologist's level of expertise and the occurrence of adverse events after botulinum toxin administration.

**Injector's Level of Expertise**	**No Adverse Event (n = 167)**	**With Adverse Event** **(n = 24)**	**OR IC95%**	**p value**
**Neurologist**	50	7	1,04 [0,41 – 2,66]	1,0
**Resident**	117	17		

A stratified analysis by type of adverse event was conducted to assess whether the frequency and magnitude of the association varied according to the professional who administered the botulinum toxin (neurologist or resident). Facial paresis was more frequent in patients treated by residents (9.7 %) compared to neurologists (3.5 %), with an odds ratio (OR) of 2.95 (95 % CI: 0.64–13.54); however, this difference was not statistically significant. In contrast, ptosis was observed more frequently in the neurologist group (5.3 %) compared to residents (0.7 %), with an OR of 0.14 (95 % CI: 0.01–1.33), suggesting a possible protective trend in the resident group, although without statistical significance. For hematoma, frequencies were similar between the two groups (3.5 % for neurologists vs. 2.2 % for residents), with an OR of 0.63 (95 % CI: 0.10–3.87), with no evidence of a significant association. See Table 5.

**Table 5:** Association between the professional's level of experience and type of adverse event.

**Type of adverse event**	**Applications by neurologist n = 57**	**Applications by residents** **n = 134**	**OR**	**IC95%**
**Facial paresis**	2(3,5%)	13(9,7%)	2,95	[0,64 – 13,54]
**Ptosis**	3(5,3%)	1(0,7%)	0,14	[0,01 –1,33]
**Hematoma**	2(3,5%)	3(2,2%)	0,63	[0,10 – 3,87]

## 4 Discussion

Although botulinum toxin is widely used and its efficacy is well established in the management of primary hemifacial spasm (HFS), there is currently little published evidence comparing the incidence of complications based on the experience level of the healthcare provider administering the toxin. Therefore, the objective of this study was to evaluate a possible association between the expertise level of neurology professionals (movement disorder specialist vs. neurology resident) and the occurrence of adverse events following the administration of botulinum toxin type A (BoNT-A).

First, this study found a cumulative incidence of adverse events of 12.6 %, a figure consistent with previous reports in the literature, where rates range between 5 % and 20 %, depending on the type of toxin, injection technique, clinical indication, and patient characteristics [12]. This analysis focused exclusively on three specific adverse events: eyelid ptosis, hematoma, and facial paresis. Due to the retrospective nature of our study, we were limited by the available data in primary medical records; however, all events were mild and transient, in line with the already well-established safety profile of botulinum toxin type A in hemifacial spasm [13].

It is worth noting that in medical settings, there is often some apprehension among patients about being treated by less experienced or younger providers. This concern relates to a widely discussed phenomenon known as the “July effect,” also referred to as the “March effect,” “August killing season,” or “Black Wednesday,” depending on the country of origin. This effect suggests a potential increase in errors and adverse events during the period when new residents and trainees begin their clinical duties [9]. However, the original study describing this phenomenon did not report a significant increase in overall healthcare costs [14]. Since then, several publications have debated the existence of this effect [9], covering multiple areas of medicine and clinical conditions such as heart failure, childbirth, stroke treatment, and spinal surgery, among others. In general, these studies have not supported the idea that care provided by trainees carries a higher risk of complications [15,16].

These findings are consistent with the results of the present study, which showed no statistically significant association between injector expertise and the occurrence of adverse events (estimated OR = 1.04; 95 % CI: 0.41–2.66); in practical terms, this suggests that the likelihood of developing adverse events after botulinum toxin administration was similar, regardless of whether the procedure was performed by a specialist with more than 10 years of experience or by a supervised neurology resident. Therefore, our results align with previously published surgical studies [17], especially considering that, to date, no comparative research specifically addressing the objectives of this study has been reported.

The lack of significant differences in the rate of adverse events between applications performed by specialists and those performed by residents suggests that botulinum toxin administration by residents is safe in controlled settings. This has important implications for clinical practice and medical education, as it supports the inclusion of supervised procedures as part of the learning curve in neurological treatment.

A considerable proportion of patients received injections from both groups (residents and specialists). However, the analysis was conducted per individual application, not per patient, allowing accurate classification of each procedure based on the responsible professional. This approach was chosen to preserve the fidelity of clinical data and ensure better estimation of the association between experience level and adverse events.

The literature reports that the most commonly treated muscles in hemifacial spasm include the orbicularis oculi, zygomaticus major, risorius, and platysma, and that the average dose per hemiface ranges between 20 and 40 units, depending on severity and prior patient response [18]. Therefore, the protocol used in our study falls within the internationally reported range and offers a conservative and standardized approach. Although dose was not analyzed as a confounding factor, its uniformity in clinical practice reduces the likelihood that it significantly influenced the occurrence of adverse events.

An important limitation is the retrospective nature of the study, which inherently limits control over data completeness and the standardization of adverse event reporting. While prospective studies would allow for more rigorous data collection, this retrospective analysis represents a valuable exploratory approach in a context with limited available evidence. All applications were performed under a standardized institutional protocol, and adverse events were systematically documented during follow-up visits.

Future prospective studies could also evaluate treatment effectiveness (spasm reduction, duration of benefit), patient satisfaction, time required to complete the procedure, and learner self-confidence. These variables are essential for designing more comprehensive training protocols aimed at improving not only procedural safety but also overall care quality and resident preparedness.

In conclusion, this study provides evidence supporting comparable safety between neurologist specialists and residents in the administration of botulinum toxin for hemifacial spasm. These findings support the incorporation of such procedures into well-structured training programs, provided that appropriate supervision is ensured.

## 5 Disclosures

- Financial Disclosures for the Previous 12 months:
- The authors declare that there are no additional disclosures to report
- Funding Sources and Conflicts of Interest:
- The authors declares that there are no conflicts of interest relevant to this work and that there are no additional disclosures to report.

## CRediT authorship contribution statement

**Melissa Luque-Llano:** Writing – original draft, Project administration, Methodology, Conceptualization. **Maria Angelica-Coronel:** Writing – original draft, Project administration, Methodology, Conceptualization. **Yulexy T. Alvarado-Vanegas:** Writing – review & editing, Writing – original draft, Conceptualization. **Floralinda García Puello:** Writing – review & editing, Supervision, Methodology. **Laura Arzuza Ortega:** Software, Methodology, Formal analysis, Data curation. **Gustavo B. Vincos:** Writing – review & editing, Validation, Supervision, Resources.

## Declaration of competing interest

The authors declare that they have no known competing financial interests or personal relationships that could have appeared to influence the work reported in this paper.
